# The Association between α-Synuclein and α-Tubulin in Brain Synapses

**DOI:** 10.3390/ijms22179153

**Published:** 2021-08-25

**Authors:** Alida Amadeo, Sara Pizzi, Alessandro Comincini, Debora Modena, Alessandra Maria Calogero, Laura Madaschi, Gaia Faustini, Chiara Rolando, Arianna Bellucci, Gianni Pezzoli, Samanta Mazzetti, Graziella Cappelletti

**Affiliations:** 1 Department of Biosciences, University of Milan, Via Celoria 26, 20126 Milano, Italy; sara.pizzi1@unimi.it (S.P.); alessandro.comincini@unimi.it (A.C.); debora.modena@unimi.it (D.M.); alessandra.calogero@unimi.it (A.M.C.); chiara.rolando@unimi.it (C.R.); samanta.mazzetti@gmail.com (S.M.); 2 Center of Excellence on Neurodegenerative Diseases, University of Milan, Via Celoria 26, 20126 Milano, Italy; 3 UNITECH NOLIMITS, University of Milan, Via Celoria 26, 20133 Milan, Italy; laura.madaschi@unimi.it; 4 Division of Pharmacology, Department of Molecular and Translational Medicine, University of Brescia, Viale Europa 11, 25123 Brescia, Italy; gaia.faustini@unibs.it (G.F.); arianna.bellucci@unibs.it (A.B.); 5 Fondazione Grigioni per il Morbo di Parkinson, Via Zuretti 35, 20125 Milano, Italy; pezzoli@parkinson.it

**Keywords:** microtubules, α-synuclein, presynaptic bouton, central nervous system, *striatum*, mouse, human brain, interaction, PLA, electron microscopy

## Abstract

α-synuclein is a small protein that is mainly expressed in the synaptic terminals of nervous tissue. Although its implication in neurodegeneration is well established, the physiological role of α-synuclein remains elusive. Given its involvement in the modulation of synaptic transmission and the emerging role of microtubules at the synapse, the current study aimed at investigating whether α-synuclein becomes involved with this cytoskeletal component at the presynapse. We first analyzed the expression of α-synuclein and its colocalization with α-tubulin in murine brain. Differences were found between cortical and striatal/midbrain areas, with *substantia nigra pars compacta* and *corpus striatum* showing the lowest levels of colocalization. Using a proximity ligation assay, we revealed the direct interaction of α-synuclein with α-tubulin in murine and in human brain. Finally, the previously unexplored interaction of the two proteins in vivo at the synapse was disclosed in murine striatal presynaptic boutons through multiple approaches, from confocal spinning disk to electron microscopy. Collectively, our data strongly suggest that the association with tubulin/microtubules might actually be an important physiological function for α-synuclein in the synapse, thus suggesting its potential role in a neuropathological context.

## 1. Introduction

α-synuclein is widely expressed in vertebrate brain and strongly implicated in various neurodegenerative disorders [1]. It started to be interesting for the scientific community in 1997, when it was disclosed as major constituent of histopathological lesions in Parkinson’s disease (PD) [2], and a point mutation in the *SNCA* gene was discovered in families with the hereditary form of this disease [3]. In healthy neurons, α-synuclein was detected in neuronal somata, dendrites and synaptic terminals of several brain regions in different species, and regional diversity in its expression was broadly reported [4,5,6,7,8,9,10], even if the use of different antibodies gave contradictory mappings and subcellular localizations [11,12]. Nevertheless, α-synuclein is mainly localized in presynaptic terminals, mostly the asymmetric excitatory glutamatergic ones [8,9], suggesting that its altered synaptic expression could be a predisposing factor for impaired high-frequency neurotransmission and for the development of several pathologies [1,13,14,15,16,17].

α-synuclein is a soluble intrinsically unfolded protein that is able to adopt multiple conformations and to interact with several partners, thus, in turn, its role in a plethora of neuronal functions is emerging [18]. Starting from the discovery that α-synuclein interacts with synaptic vesicles [19] and promotes the assembly of the vesicular SNARE complex [20], many studies added pieces to the role of α-synuclein at the synapse over time and disclosed its implication in synaptic vesicle function and recycling. In detail, α-synuclein promotes clustering of synaptic vesicles [21,22], inhibits docking [23], and regulates the activity of dopamine transporters [24]. In addition, α-synuclein interacts with the vesicular monoamine transporter VMAT-2 [25] and synapsin III [26], regulating synaptic function in dopamine neurons. However, the contribution of α-synuclein to neurotransmission under physiologically relevant conditions proved to be elusive, even if a very recent paper strongly demonstrated that α-synuclein plays a dual role in both the facilitation and depression of dopamine release in vivo [27].

The cytoskeleton was reported to be a cellular partner of α-synuclein. It is a matter of debate as to whether and how this interplay could be pivotal for synaptic function. Focusing on the actin cytoskeleton, evidence exists that α-synuclein binds actin and regulates its dynamics [28], possibly tuning the vesicle release process [29]. The interplay with tubulin is actually more controversial [30,31]. The binding of α-synuclein to tubulin and the impact on tubulin polymerization, although unclear, was disclosed in vitro by multiple approaches including co-immunoprecipitation [32,33], fibrillogenesis and immunoelectron microscopy [34], colocalization [35], microtubule assembly assays [36] and live-cell imaging [37]. This interaction was also underlined in vivo by colocalization and immunoelectron microscopy [6,33,37], and by electrophysiological recordings and pharmacological treatments [15,16]. Nevertheless, there are several hints highlighting that α-synuclein might have a role in modulating the microtubular dynamics. Recently, we found that α-synuclein folds upon interaction with tubulin and pointed out its novel role in regulating multiple steps of microtubule dynamics in cell-free systems and in neuronal cells [38]. Despite this, to date, there have been no investigations aimed at elucidating the potential interaction of α-synuclein and tubulin in vivo at the synapse. One major doubt regarding the relevance of the interplay of α-synuclein with tubulin for synaptic function comes from the old view that synaptic vesicles in presynaptic terminals are not in contact with microtubules. Indeed, the presence of tubulin/microtubules in synaptic terminals was reported not only in vitro [39,40,41], but also in vivo [42,43,44,45,46], both in invertebrate and rodent models. Importantly, it is now clear that microtubule dynamics play a fundamental role in neurotransmission, controlling vesicle motility at presynaptic boutons [47] and regulating synaptic vesicle cycling [48,49].

Given this emerging role of microtubules at the synapse, unravelling the role of the interaction between α-synuclein and tubulin in this neuronal compartment remains a crucial challenge. Here, we firstly focused on α-synuclein expression in specific areas of wild-type murine brain, using biochemical and immunohistochemical methods, to give an overview of its distribution in specific brain areas. Afterwards, we deeply investigated the interplay between α-synuclein and α-tubulin using high-level and original morphological approaches, including the Proximity Ligation Assay (PLA) and ultrastructural analysis, with a focus on *corpus striatum* synapses. Moreover, we also validated their relationship in post-mortem human brain.

## 2. Results

### 2.1. α-Synuclein Distribution Changes in Different Areas of Murine Brain

**Figure 1:**
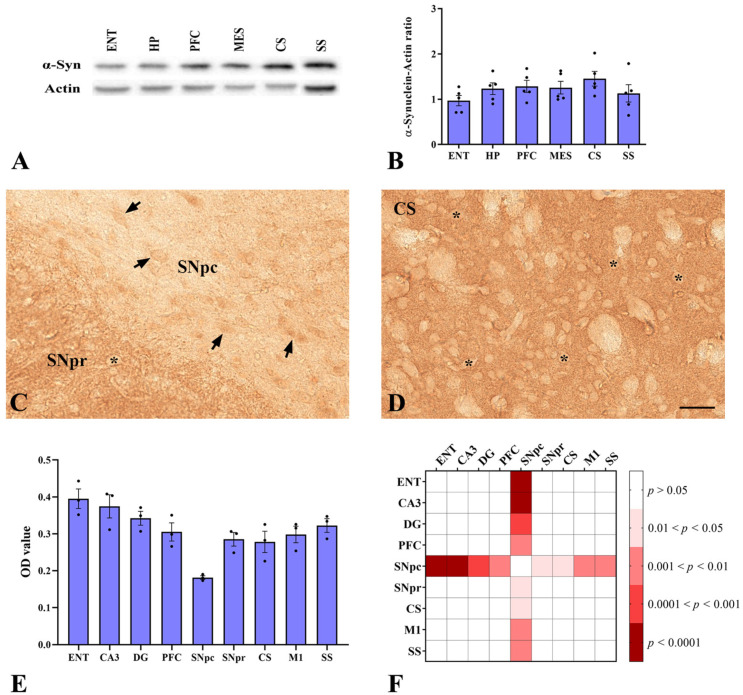
α-synuclein expression in WT murine brain. (**A**,**B**): Western blot analysis of α-synuclein levels in total lysates obtained from different cerebral regions. (**A**): Representative western blot showing α-synuclein levels in total lysates of entorhinal cortex (ENT), hippocampal formation (HP), prefrontal cortex (PFC), ventral mesencephalon (MES), *corpus striatum* (CS), and somatosensory cortex (SS). Actin is used as loading reference. (**B**): Quantification of α-synuclein protein levels normalized to actin. Data are mean ± SEM (*n* = 5 mice); One-way ANOVA was used for statistical analysis followed by Bonferroni’s multiple comparisons test. (**C**,**D**): α-synuclein immunoperoxidase staining in coronal sections of WT mice brain. Light microscope images of α-synuclein immunolabeling in the *substantia nigra pars compacta* (SNpc) and *reticulata* (SNpr; (**C**)) and *corpus striatum* (CS; (**D**)). α-synuclein staining is punctiform and mainly neuropilar. The asterisks showed that the cell bodies are not labelled, except in the SNpc (arrows). Scale bar: 40 µm. (**E**): Densitometric analysis of α-synuclein immunoperoxidase staining in different areas of WT mice brain. Data are shown as mean optical density ± SEM (*n* = 3 mice). One-way ANOVA was used for statistical analysis followed by Bonferroni’s multiple comparison test as described in the Materials and Methods section. *p* values are graphically represented in the heat-map in (**F**) (P60, *n* = 3). CA3: *Cornu Ammonis* region 3; DG: dentate gyrus; M1: primary motor cortex.

We first evaluated the expression of α-synuclein protein in the forebrain and midbrain areas of adult wild-type (WT) mice, at postnatal day 60 (P60), by Western Blotting (Figure 1A) and densitometric analysis (Figure 1B). Although the differences were not significant, we found that the maximal expression of α-synuclein was observed in *corpus striatum*, while the entorhinal cortex showed the lowest amount, suggesting that α-synuclein expression could be area-specific in the murine brain. Thus, we proceeded to investigate, in detail, the distribution of α-synuclein through an immunohistochemical approach. According to the prevalent localization at the presynaptic site reported in the literature [5,8], staining for α-synuclein appeared mainly in the neuropil and scarcely ever in neuronal cell bodies. However, in the *substantia nigra pars compacta*, the labelled puncta were sparse and the product of the reaction was mainly detected in the soma of some neurons (Figure 1C). Conversely, the *striatum* (Figure 1D) and somatosensory/entorhinal cortices (Figure S1A,D) showed a broadly diffuse and punctiform staining surrounding negative neuronal cell bodies. Interestingly, an intense labelled band between the granule cell and molecular layers of dentate gyrus (Figure S1B), and between the pyramidal cell and molecular layers of CA3 (*Cornu Ammonis*, region 3) *stratum oriens* (Figure S1C), was observed. The densitometric analysis performed on these brain areas strongly suggests a region-dependent distribution of α-synuclein. As shown in Figure 1E,F, some striking differences among areas were detectable. In detail, the expression level of the protein in the *substantia nigra pars compacta* appeared significantly lower than in the hippocampus (CA3 and dentate girus) and all the cortical regions we analyzed (Figure 1F).

To also check the subcellular distribution of α-synuclein, we performed an ultrastructural analysis on the prefrontal cortex, *corpus striatum* and *substantia nigra pars compacta* (Figure S2), confirming the predominant presynaptic localization of the protein in accordance with previous ultrastructural studies [5,6]. No glial structures showed α-synuclein staining (Figure S2). The subcellular localization via the immunoperoxidase ultrastructural method was confirmed with a single immunogold reaction during pre-embedding (not shown), as fully illustrated by the double immunogold experiments reported below.

### 2.2. α-Synuclein and α-Tubulin Colocalize in the Brain at the Presynapse

**Figure 2:**
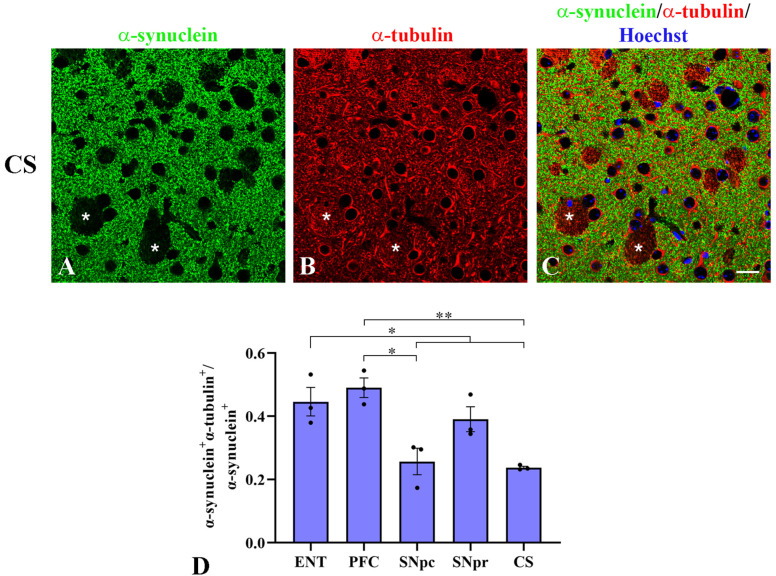
α-synuclein and α-tubulin colocalization in different brain areas of WT mice. (**A**–**C**): Double immunofluorescence in *corpus striatum* (CS) of WT mice for (**A**) α-synuclein (α-syn, green) and (**B**) α-tubulin (α-tub, red), and (**C**) the relative merge with Hoechst in blue as nuclear staining. Asterisks indicate bundles of fibers. (**D**): Colocalization analysis between α-synuclein and α-tubulin. The bars represent the Manders’ coefficient 1 (M1) computed for each brain area; data are shown as mean ± SEM (*n* = 3 mice); one-way ANOVA was used for statistical analysis followed by Bonferroni’s multiple comparison test as described in the Materials and Methods section; * *p* ≤ 0.05, ** *p* ≤ 0.01. ENT: entorhinal cortex; PFC: prefrontal cortex; SNpc: *substantia nigra pars compacta*; SNpr: *substantia nigra pars reticulata*; CS: *corpus striatum*. Scale bars: 20 µm.

Given the emerging data on the interplay between α-synuclein and α-tubulin in vitro [32,34,37,38], we wondered whether this interaction occurs in vivo. Firstly, colocalization analysis on double immunofluorescence was carried out in those representative murine brain areas (*corpus striatum*, *substantia nigra pars compacta and reticulata*, entorhinal and prefrontal cortices) that are homologous to regions prone to α-synuclein aggregation and Lewy body formation in humans [50]. We observed the overall colocalization of α-synuclein and α-tubulin in neuronal cell bodies, processes and puncta (Figure 2A–C). The degree of overlap was defined by the M1 Manders’coefficient, which represents a good indicator of the α-synuclein fraction coincident with α-tubulin. As reported in Figure 2D, the different brain regions displayed several degrees of colocalization, all of them lower than 50% of the total α-synuclein. Statistically significant differences were observed between prefrontal and entorhinal cortices versus subcortical areas including both the *substantia nigra pars compacta* and *striatum* (Figure 2D). We observed that the degree of α-synuclein/α-tubulin colocalization followed the same trend of α-synuclein levels in the examined areas. Basically, they were both higher in the cortical areas and lower in the nigrostriatal system.

**Figure 3:**
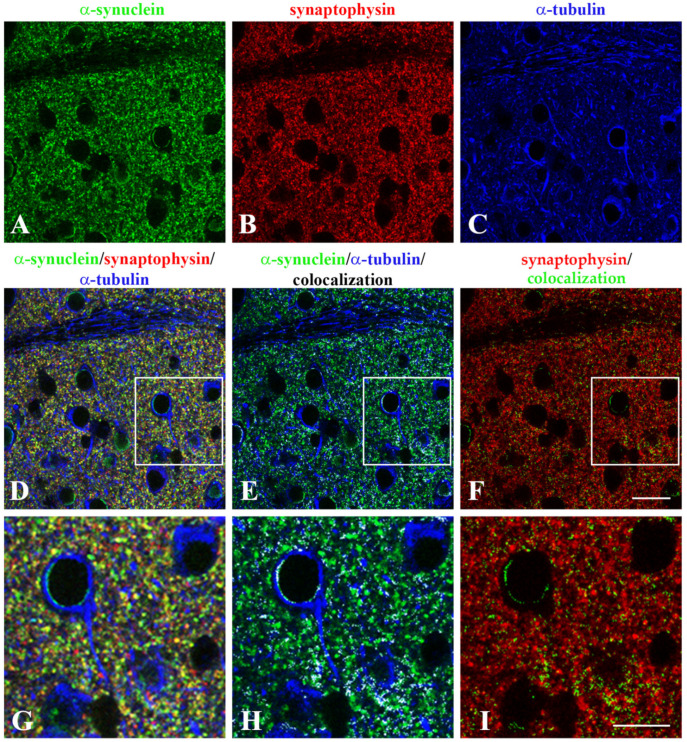
α-synuclein and α-tubulin colocalization in synaptic terminals in the *corpus striatum* of WT mice. Triple immunofluorescence for (**A**) α-synuclein (α-syn, green), (**B**) synaptophysin (syp, red), (**C**) α-tubulin (α-tub, blue) analyzed by confocal microscopy (*n* = 4 mice, at least 4 replicates). (**D**): Merge of the triple staining. The region marked by the white square in (**D**) is magnified in (**G**). (**E**): The colocalization between α-synuclein (green) and α-tubulin (blue) is shown as the white mask. The region marked by the white square in (**E**) is magnified in (**H**). (**F**): Merge of synaptophysin (red) with the α-synuclein/α-tubulin colocalization mask (green). The region marked by the white square in (**F**) is magnified in (**I**). Scale bars: 25 µm (**A**–**F**), 40 µm (**G**–**I**).

Then, we focused on synapses to obtain an insight into the neuronal compartment where the interplay between α-synuclein and α-tubulin might occur. The analysis was carried out on *corpus striatum*, where a huge amount of synaptic terminals arising from different sources interacts in a neurochemically complex neuropil [51,52]. Notably, this region is involved in the signal transmission from the neocortex and receives afferent pathways from the *substantia nigra*, giving it a crucial role in studies concerning PD. To achieve our goal, we firstly confirmed the presence of α-tubulin in striatal synapses by ultrastructural analysis after pre-embedding immunogold staining (Figure S3). Then, we carried out a triple immunofluorescence for α-synuclein, α-tubulin and synaptophysin, which is a marker of the pre-synaptic compartment (Figure 3A–D,G). We evaluated α-synuclein/α-tubulin colocalization (Figure 3E,H) and combined α-synuclein/α-tubulin colocalization with synaptophysin (Figure 3F,I) in *corpus striatum*. To obtain quantitative outcomes from this analysis, we selected α-synuclein/α-tubulin colocalization signal (white in Figure 3E,H), overlapped this image mask (green in Figure 3F,I) with synaptophysin, and performed colocalization analysis between the three antigens. A mean M2 Manders’ coefficient of 0.168 revealed that almost 17% of the total synapses in *corpus striatum* contained both α-synuclein and α-tubulin (yellow signal in Figure 3F,I). Taken together, these results demonstrated a considerable colocalization of α-synuclein/α-tubulin that varies in selected forebrain and brainstem areas, and revealed that such a colocalization between the two proteins occurs in a subset of synaptic terminals in *corpus striatum*.

### 2.3. α-Synuclein and α-Tubulin Interact in Mouse Corpus Striatum and in Human Brain

**Figure 4:**
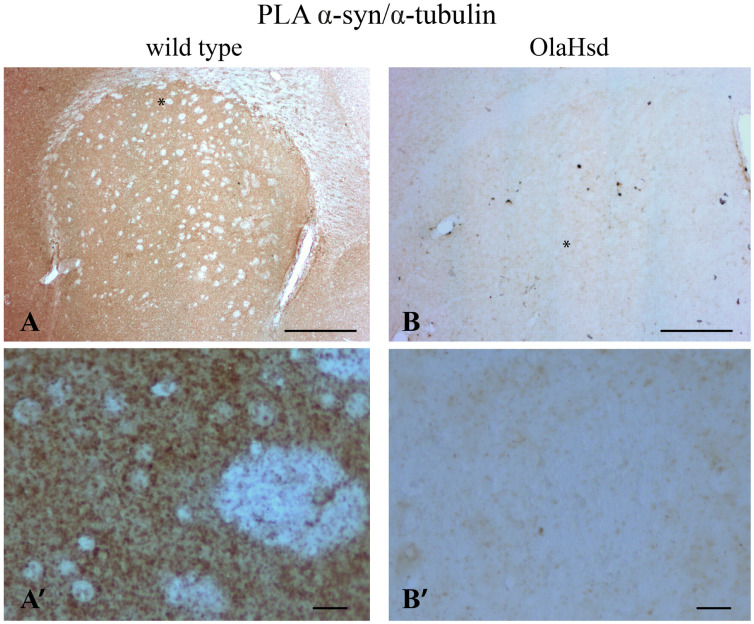
Brightfield PLA procedure for α-synuclein/α-tubulin PLA in *corpus striatum* of wild type and OlaHsd mice. (**A**,**B**): Low magnification image of PLA staining (brown) for α-synuclein/α-tubulin PLA in wild type mouse brain *corpus striatum* ((**A**), *n* = 4 mice) compared to the almost negative signal in OlaHsd mouse ((**B**), *n* = 4 mice). In (**A**), white dots are the typical bundles of fibers in the *striatum* that are negative for the PLA staining. (**A’**,**B’**): High magnification photomicrographs of the *striatum*. Asterisks in (**A**,**B**) indicate the regions that are magnified in (**A’**,**B’**), respectively. Scale bars: 500 µm (**A,B**), 10 µm (**A’**,**B’**).

**Figure 5:**
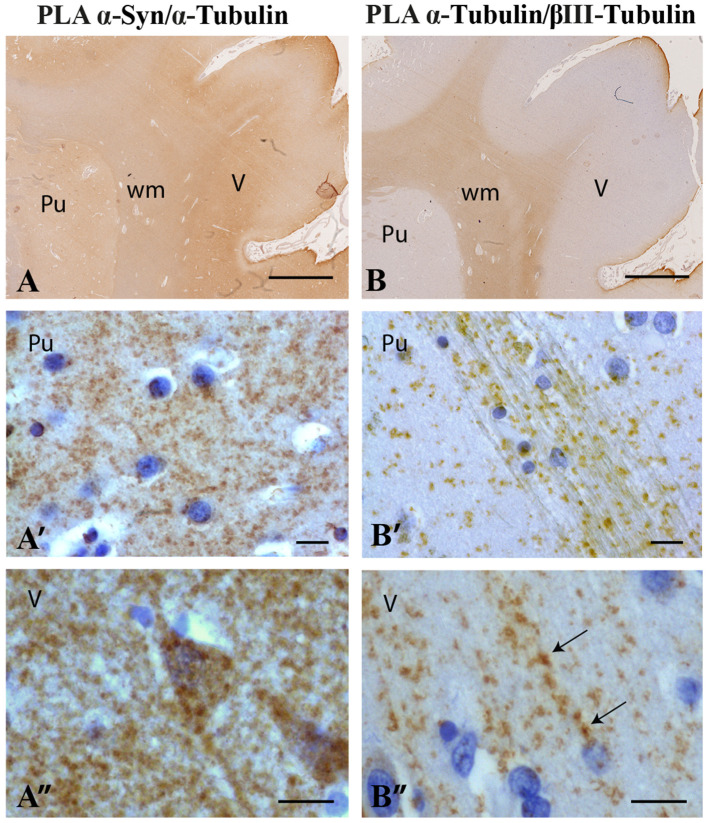
Brightfield PLA procedure for α-synuclein/α-tubulin PLA in human *corpus striatum* and cerebral cortex. (**A**): Low magnification image of PLA staining (brown) for α-synuclein/α-tubulin PLA in human brain *corpus striatum* and cerebral cortex (*n* = 4 subjects). (**A’**,**A”**): High magnification photomicrographs of putamen (Pu, (**A’**)) and cerebral cortex layer V (V, (**A”**)). (**B**): Low magnification image of PLA staining (brown) for α-tubulin/βIII-tubulin PLA in human *corpus striatum* and cerebral cortex used as a positive control. (**B’**,**B”**): High magnification photomicrographs of putamen (**B’**) and cerebral cortex layer V (**B”**). Arrows indicate signal in the apical dendrites of pyramidal neurons. Nuclei are counterstained with hematoxylin (violet). wm: white matter. Scale bars: 2.5 mm (**A**,**B**), 20 µm (**A’**–**B”**).

Once the colocalization of α-synuclein and α-tubulin was revealed in the synaptic compartment, we decided to investigate whether the two proteins interact by means of brightfield PLA in both murine (Figure 4A,A’) and human (Figure 5A,A’) *corpus striatum*. First, we observed the association of the two proteins in murine brain (Figure 4A,A’). The specificity of the PLA technique was assessed by negative controls using two approaches: (i) omitting anti-α-synuclein primary antibody from PLA, and (ii) testing α-synuclein/α-tubulin PLA on sections of C57BL/6J OlaHsd mice carrying spontaneous deletion of the *SNCA* gene [53]. As expected, no staining was present with the omission of the primary antibody (Figure S4) and no specific signal was detected in the C57BL/6J OlaHsd mouse sections where α-synuclein is not expressed (Figure 4B,B’). Second, we found that α-synuclein also interacts with α-tubulin in the human brain (Figure 5A,A’). Here, we observed a diffused signal mostly in the grey matter in the *corpus striatum* (Figure 5A,A’) and also in the cerebral cortex, especially in layer V (Figure 5A,A”), where a high synaptic density is observable. As a positive PLA control, we assessed the α-tubulin/βIII-tubulin interaction in the human brain sample and reported an expected pattern of staining that was more intense in the white matter (Figure 5B,B’), showing its specificity for regions that were rich in microtubules in the bundles of fibers of the *corpus striatum* (Figure 5B’) or in the microtubules present in the apical dendrites of the pyramidal neurons (layer V, Figure 5B”).

**Figure 6:**
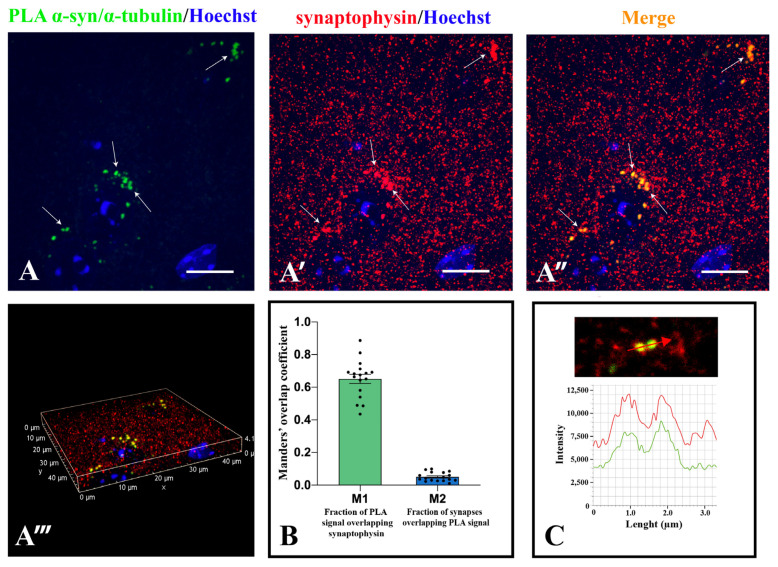
Spinning disk analysis of α-synuclein/α-tubulin interaction in the synapses of murine *corpus striatum*. (**A**): Representative maximum projection of α-synuclein/α-tubulin PLA staining (green, (**A**,**A’’**)) and synaptophysin staining (red, (**A’**,**A’’**)) in C57BL/6J mouse *corpus striatum*. White arrows point to some of the α-synuclein/α-tubulin PLA signals that colocalize with synaptophysin (yellow dots also in **A’’’**,**C**). Nuclei are counterstained with Hoechst (blue). Scale bars: 5 µm (**A**–**A’’**). (**A’’’**): 3D reconstruction of all the immunofluorescence signals contained in the tissue slice is showed in the maximum projection. (**B**): M1 and M2 Manders’ overlapping coefficient obtained from 16 images acquired from 4 different mice. (**C**): Intensity profile plot representing the values indicated by the red arrow in the single stack image of the merge channels stained for synaptophysin (red) and α-synuclein/α-tubulin PLA (green).

Based on the evidence that α-synuclein and α-tubulin directly interact in mouse and human brain, we analyzed whether this interaction occurs in the synaptic compartment. We performed fluorescent PLA experiments in combination with synaptophysin immunofluorescence in order to detect the interaction between the two proteins in striatal synaptic terminals (Figure 6). The PLA signal (Figure S5A and Figure 6A) colocalized with synaptophysin labelling (Figure S5 and Figure 6A’) in the merge images (Figure S5 and Figure 6A’’). In detail, the fraction of PLA signal colocalizing with synaptophysin was more than half (obtained by Manders’ coefficients, M1 = 0.65). Furthermore, only a small percentage (5%) of striatal synaptic terminals (M2 = 0.05) contained the association between the two proteins (Figure 6B). Then, analysing the intensity profiles (Figure 6C), we confirmed a complete superimposition of the α-synuclein and α-tubulin staining spectrum and the synaptophysin one, indicating that this interaction really occurred in some synapses.

**Figure 7:**
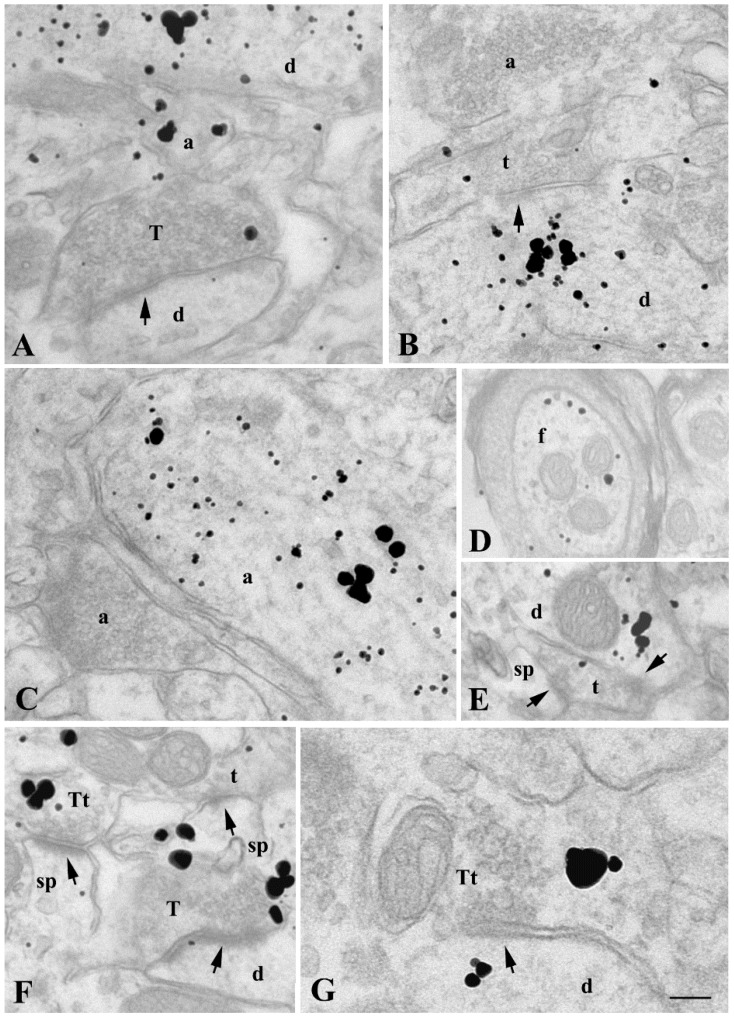
Immunoelectron microscopical localization of α-synuclein and α-tubulin in murine *corpus striatum*. Representative electron micrographs of α-synuclein (large silver-intensified gold particles) and α-tubulin (small silver-intensified gold particles) pre-embedding immunogold colocalization performed in C57BL/6J mice (*n* = 3). The colocalization appeared mainly in proximal dendrites (d), as shown in (**A**,**B**,**E**), as well as in some, but not all, small and large vesicle-containing axonal (a) profiles (**A**,**C**). Some poorly myelinated fibers (f in (**D**)) and small axonal terminals forming synaptic contacts (arrows) with proximal dendrites (d) and dendritic spines (sp) displayed single α-tubulin immunolabelling (t in (**B**,**E**,**F**)), whereas single α-synuclein immunolabelled synaptic boutons (T) contacting (arrows) distal dendrites (d) were shown in (**A**,**F**). Finally, double α-synuclein and α-tubulin immunogold staining was displayed by some small synaptic terminals (Tt) forming either asymmetric postsynaptic specializations (arrows in (**F**)) on dendritic spines (sp) or symmetric synapses (arrow in (**G**)) with α-tubulin-positive dendrites (d in (**G**)). Scale bar: 200 nm (**A**–**F**), 100 nm (**G**).

Finally, we investigated the interaction of α-synuclein with α-tubulin inside the synapse at the ultrastructural level by carrying out a double immunogold pre-embedding localization. This technique enabled us to observe the presence of both antigens in the neuropilar structures of *corpus striatum* (Figure 7). The discrimination of intensified signals of α-synuclein and α-tubulin immunogold silver was possible through the double silver intensification of α-synuclein-related gold particles, which made them larger than the α-tubulin-related ones. First of all, the single immunolocalization of α-synuclein in some synaptic boutons (Figure 7A,F), and of α-tubulin in partially myelinated fibers (Figure 7D) and in some synaptic terminals (Figure 7B,E,F), reasserted our previous single ultrastructural immunolabelling (Figures S2 and S3) and gave us a validation of the double immunogold method. Moreover, our analysis confirmed the colocalization of the two proteins in dendritic shafts (Figure 7A,B,E,G) as well as in small (Figure 7A) and large (Figure 7C) axons. Finally, in the striatal neuropil, we identified a few small synaptic terminals, which contacted dendrites or dendritic spines and clearly displayed both α-synuclein- and α-tubulin-related gold particles, among or adjacent to small clear vesicles (Figure 7F,G).

In conclusion, our data demonstrated a strict relationship between α-synuclein and α-tubulin not only in different neuronal compartments such as dendrites and axons, but also in synapses where their mutual interaction could be crucial for neurotransmission.

## 3. Discussion

Since α-synuclein was indicated as the major component of histopathological lesions in a spectrum of neurodegenerative diseases including PD, many studies were centered on its propensity to aggregate and form pathological inclusions [54], thus leaving its physiological role mainly elusive. Here, we addressed the emerging issue of the interplay of α-synuclein with microtubules. Beyond the differential expression of α-synuclein and its colocalization with α-tubulin in some areas of the murine brain, our data unravel the previously unexplored association of the two proteins that may be relevant for synaptic function in vivo. Given the impact of α-synuclein on the dynamic instability of microtubules in cell-free systems and neuronal cultured cells [38], the present work paves the way for investigating a novel physiological role of α-synuclein in the regulation of microtubule behavior at the synapse that, in turn, could help in the full comprehension of synaptic dysfunction occurring in both neurodegenerative and neurodevelopmental disorders.

The interaction of α-synuclein with tubulin and microtubules has been a matter of debate for a long time, mainly due to its potential implication in the pathogenesis of synucleinopathies [31,55,56]. However, the majority of data come from in vitro and cultured cell studies that were focused on understanding the consequences of their interplay on the biochemical properties of the two partners [30], microtubule dynamics [38] and axonal transport [37]. Nevertheless, few data are available regarding α-synuclein colocalization with tubulin in vivo. The presence of α-tubulin in Lewy bodies, pale bodies and Lewy neurites was highlighted by Alim et al. [33] in human brain affected by PD and other synucleinopathies, whereas a proximity of α-synuclein with axonal microtubules was observed exclusively by immunogold localization in cortical rat brain tissue [6]. Here, we reveal, for the first time, the colocalization of α-synuclein with microtubules in different areas of the murine brain, offering an overview on the regional differences. The highest levels of colocalization were found in the analyzed cortical areas, whereas both the *substantia nigra pars compacta* and the *corpus striatum* displayed low colocalization values in the total α-synuclein expression. In the *corpus striatum,* which is a focal structure that is rich in synaptic contacts originating from different brain areas (i.e., the cerebral cortex, brainstem and thalamus), we demonstrated the interaction of α-synuclein with α-tubulin using a PLA approach. Finally, we validated this interaction in human brain sections, not only in *corpus striatum*, which is clearly involved in PD and multiple system atrophy, but also in the cerebral cortex, a region that is relevant for dementia with Lewy bodies. Thus, our results point out α-tubulin as a novel interactor of α-synuclein, suggest that the colocalization of the two proteins is region-specific, and pose the question as to whether this could reflect a functional peculiarity.

The regional specificity in the colocalization could go together with the differential expression of α-synuclein in the brain as this work and previous papers demonstrate. Although no region-dependent changes were revealed by immunoblotting assays on brain lysates, the detailed analyses of brain areas by immunohistochemistry revealed significant alterations in the distribution and expression levels of α-synuclein in the forebrain (especially in distinct cortical areas) and ventral mesencephalon. Previous data based on semiquantitative analysis in the rodent brain suggest that many differences occur in the olfactory bulb, brainstem, thalamus, cerebellum, hippocampus, amygdala, *striatum* and neocortex [4,7,8,11]. Here, we moved to the first quantitative rating on α-synuclein distribution and revealed significant differences between entorhinal cortex/CA3 and other cortical and striatal areas. In particular, *substantia nigra pars compacta* appeared to be the least labelled area, showing a significant difference to all the other examined regions. Focusing on the midbrain region, an interesting scenario emerged from the analysis of α-synuclein distribution together with its colocalization with α-tubulin, which was completely unexplored until now. Beyond the differences observed in data coming from different laboratories that could depend on differences among antibodies, which is an important issue, as previously discussed [12], a common finding between ours and other papers [4,8] is the detection of somatic α-synuclein almost exclusively in the *substantia nigra pars compacta*. Collectively, these results suggest that the differences in α-synuclein distribution inside neuronal compartments and protein interaction could mark this brain area and impose peculiar behavior in physiology, and perhaps in pathology.

The finding that α-synuclein interacts with tubulin at the synapse is groundbreaking as it sheds light on the emerging role of microtubules in this neuronal compartment. The presence of tubulin and microtubules in the synapse is a long-lasting controversial issue [57]. Even if, on the one hand, microtubules are easily visualized in axons and dendrites through multiple classical techniques, on the other hand, their synaptic localization has always been difficult due to their instability and scarcity in this neuronal compartment. Indeed, a long time ago, Gordon-Weeks et al. [42] demonstrated the presence of presynaptic microtubules in central synapses, autonomic varicosities, and also rat brain synaptosomes, at the ultrastructural level, by using specific fixatives enriched in EGTA, which avoided their disassembly by calcium influx. However, it was more than three decades later when evidence from live cell imaging experiments in *Drosophila* models definitively concluded that microtubules are present inside synaptic boutons [45]. More interestingly, recent studies in mammalian neurons demonstrated the role of microtubules in the release of synaptic vesicles [58]. Microtubule dynamics is crucial in excitatory en passant varicosities where they are able to correctly target synaptic vesicles to active zones, thus controlling neurotransmission [47]. In this scenario, the results we obtained through multiple approaches, from PLA and confocal spinning disk microscopy to double immunogold pre-embedding and electron microscopy, supply a novel insight into presynaptic functions. So, we speculate that the presynapse could be the privileged compartment where α-synuclein modulates synaptic release and activity via the regulation of microtubules due to its dynamase activity [38].

Our finding showing a direct interaction of α-synuclein and α-tubulin in a subset of striatal murine synapses strongly suggests that this could mark specific synapses. Interestingly, α-synuclein is mainly expressed in asymmetric synapses, generally excitatory, in the *corpus striatum* [5]. In parallel, a high grade of colocalization between α-synuclein and the vesicular glutamate transporter-1 (VGLUT1, a marker of most glutamatergic terminals) and the low colocalization with GAD67 (a GABAergic marker) were detected in the cerebral cortex [8]. Now, the challenge is to better understand if and how the expression of α-synuclein modulates the activity of specific synaptic terminals with different neurochemical properties and whether its interaction with α-tubulin plays a role. Indeed, based on the most recent studies on synaptic microtubules [49,57] and on our results, en passant synapses could represent the best example of sites where α-synuclein and α-tubulin might interact and cooperatively impact on synaptic activity. In this context, old data reporting ultrastructural characterization of dopaminergic striatal innervation revealed its en passant distribution in rat *striatum* [59]. On the other hand, we observed a high level of the two proteins colocalization in cortical areas that are notably rich in modulatory cholinergic, dopaminergic and serotoninergic en passant varicosities [60,61,62]. Lastly, studies regarding the modulatory role and localization of α-synuclein in brain synapses were mainly focused on excitatory glutamatergic synaptic contacts [8,9,16,63], where they could interact to orchestrate a fine tuning of glutamate release by specialized hotspots, as recently stated by Qu et al. [47] in hippocampal cell cultures. As a whole, these studies lay the foundation for further analyses designed to better understand the role of the α-synuclein/α-tubulin interplay in synaptic terminals that possess different neurochemical properties, where they could interact to orchestrate the fine tuning of neurotransmitter release.

Our data, which point to a novel aspect of α-synuclein biology (specifically, its interaction with tubulin at the synapse), open an interesting scenario to explain disease mechanisms in synucleinopathies and other disorders. One initial consideration is based on the “hub protein” role of α-synuclein that refers to its propensity to interact with multiple partners at the synapse and, in turn, to regulate neurotransmission [64]. Disruption of this complex network, even when just one partner is altered, triggers synaptic dysfunction. Notably, we know that α-synuclein mutants linked to familial PD impact the tubulin system at different levels including impairment of tubulin polymerization [31,34,38], and that overexpression of α-synuclein disrupts the microtubule network [65], thus suggesting that defects in the interplay between α-synuclein and tubulin could be detrimental for neurons and lead to neurodegeneration. Furthermore, in a murine model of multiple system atrophy, α-synuclein/β-III tubulin protein complex was involved in synaptic vesicle release and reduces GABAergic inhibitory neurotransmission [15]. A second line of evidence comes from studies on the aggregation of α-synuclein. Oligomeric α-synuclein inhibits tubulin polymerization [35], whereas the modulation of tubulin system is effective in blocking the formation of pathological α-synuclein inclusions [66]. On these bases, our findings could suggest new investigations on the α-synuclein/α-tubulin interplay, not only in classical synucleinopathies but also in other neuropathologies such as Alzheimer’s disease and epilepsy where synaptic dysfunction frequently underlies the respective clinical pictures [57]. Interestingly, studies on neurodevelopmental diseases point out the role of both deletion or partial duplication of the α-synuclein gene [67] and genetic mutations in microtubule-associated genes or defective regulation of microtubules in the pathophysiology of autism spectrum disorder [68]. This could permit speculation on the existence of a converging mechanism that involves both α-synuclein and tubulin cytoskeleton in neurodegenerative as well as neurodevelopmental disorders.

To conclude, our demonstration of the association between α-synuclein and α-tubulin in murine and human brain tissues proposes a new angle for looking at synaptic compartment and machinery in neuronal health, but also in disease.

## 4. Materials and Methods

Twelve male WT C57BL/6J mice at P60 were purchased from Charles River (Calco, Italy) and used for all experiments. The mice were maintained in pathogen-free conditions and bred with free access to water and standard pelleted diet. The mice were killed by decapitation or by intracardiac perfusion, to perform biochemical or immunohistochemical analysis, respectively. All procedures were compliant to Italian law (D. Lgs 2014/26, implementation of the Directive 2010/63/UE of the European Parliament and of the Council on the protection of animals used for scientific purposes) and approved by the University of Milan Animal Welfare Body and by the Italian Ministry of Health (project authorization number: 901/2015-PR).

Four C57BL/6J OlaHsd mice, a substrain of C57BL/6J mice carrying a spontaneous deletion of the SNCA gene, were stored at the University of Brescia and all procedures were approved by the Italian Ministry of Health (project authorization number: 719/2015-PR).

### 4.1. Human Samples

Formalin fixed paraffin embedded striatal sections were obtained from post-mortem human brains of four control subjects, including three females (64, 82, 93 years old) and one male (71 years old), in whom the absence of neurodegenerative pathologies was assessed (see [69] for details). Written informed consent was obtained from all subjects in compliance with relevant laws and institutional guidelines and approved by the appropriate committees.

### 4.2. Primary Antibodies

All primary antibodies, their epitope, host species, application dilution, source and catalogue number are summarized in Table 1.

### 4.3. Western Blot Analysis

Western blot analysis was performed on protein extracts obtained from brain regions of five mice. To obtain total proteins, *corpus striatum*, ventral midbrain, hippocampus, entorhinal, prefrontal and parietal cortices were immediately dissected on ice and mechanically homogenized and sonicated in sample buffer (SB1x: 2% SDS, 10% glycerol, 5% β-mercaptoethanol, 0.001% bromophenol blue and 62.5 mmol/L Tris, pH 6.8), containing protease and phosphatase inhibitors (Protease Inhibitor Cocktail, P8340 Sigma-Aldrich, St. Louis, MO, USA; Phosphatase Inhibitor Cocktail Set V, 524629, Millipore, Burlington, MA, USA). After centrifugation, soluble fractions were collected and protein concentration was measured with Pierce BCA protein Assay Kit (Thermo Fisher Scientific, Waltham, MA, USA). Equal amounts of each sample were separated by SDS-PAGE and blotted onto PVDF membranes (Immobilon^TM^-P, Millipore). Membranes were incubated for 30 min (min) at room temperature with 0.4% paraformaldehyde in PBS and then blocked with 3% bovine serum albumin (BSA) in 0.05% Tween20 Tris buffered saline (TBS-T) for 1 h (h) at room temperature and probed with the following antibodies: anti actin IgG (A2066) and anti α-synuclein (S3062). Membranes were washed for 30 min with TBS and incubated for 1 h at room temperature with HRP goat anti-rabbit IgG (1:4000; Cell Signaling Technology, Danvers, MA, USA). Chemiluminescent signals were detected using the Supersignal West Pico Chemiluminescent Substract kit (Pierce, Appleton, WI, USA). Acquisition and quantification were performed by ChemiDoc and Image Lab software (Bio-Rad, Hercules, CA, USA).

### 4.4. Immunohistochemistry

Mice were anesthetized with isoflurane and intraperitoneal 4% chloral hydrate (2 mL/100 g) and sacrificed by intracardiac perfusion as previously described [70]. Brains were immersed in 4% paraformaldehyde in phosphate buffer 0.1 M (PB), for 24 h at 4 °C. Next, brains were stored in PB, since they were cut in serial coronal brain sections (50 µm thick) with a VT1000S vibratome (Leica Microsystems). After intracardiac perfusion C57BL/6J OlaHsd mice brains were post-fixed for 2 h in 4% paraformaldehyde and conserved in 18% sucrose solution in phosphate buffered saline 0.01 M (PBS). The brains were then cut in 25 μm coronal sections with a cryostat and conserved in 60% glycerol.

According to Franklin and Paxinos [71], we chose the following sections for immunohistochemistry and densitometric analyses: (i) prefrontal cortex between +2.58 and +1.14 mm from bregma (containing secondary motor region, also known as M2, or Fr2); (ii) somatosensory cortex and *corpus striatum* in proximity to −0.34 mm from bregma; (iii) *substantia nigra pars compacta* and *substantia nigra pars reticulata* at −3.64 mm from bregma; (iv) the entorhinal cortex and the hippocampus at −3.16 mm from bregma.

#### 4.4.1. Immunoperoxidase Procedure

After aldehyde quenching with NH_4_Cl (0.05 M in PBS) for 30 min and inactivation of endogenous peroxidases with 1% H_2_O_2_ (in PBS) for 30 min, sections were permeabilized with a mild pretreatment by ethanol (10%, 25%, 10% in PBS, 5 min each) to increase the immunoreagent penetration. The blocking solution constituted by 0.1% Triton X-100 in 1% BSA in PBS was then applied for 30 min. Next, slices were incubated overnight with anti-α-synuclein antibody (S3062) diluted in 0.1% BSA, at room temperature. This procedure was followed by incubation with biotinylated goat anti-rabbit IgG (1:200; Vector Laboratories, Burlingame, CA, USA), for 75 min. After washing, sections were treated with the avidin-biotin complex (ABC elite kit; Vector Laboratories; diluted 1:100) and then with a freshly prepared solution (0.075%) of 3-3′-diaminobenzidine tetrahydrochloride (Carl Roth GmbH & Co., Karlsruhe, Germany) and 0.002% H_2_O_2_. Finally, sections were mounted, dehydrated with ethanol (75%, 96% and 100% for 5 min each), immersed in xylene for 10 min and laid on coverslips with Eukitt^®^ (O. Kindler GmbH, Freiburg, Germany). The specificity of primary antibodies was assessed by negative controls, e.g., omission of primary antiserum. In these cases, no specific staining was ever observed.

#### 4.4.2. Densitometric Immunoperoxidase Analysis

To perform a densitometric analysis of α-synuclein on immunoperoxidase-stained sections, whole slices were acquired with the Nanozoomer S60 slide scanner (Hamamatsu, Tokyo, Japan). The images were then magnified and saved at 20× with the NDPview2 software (Hamamatsu) to show only the considered area. Different regions of interest (ROIs) were drawing for each brain areas using Fiji. The following areas were analyzed: prefrontal cortex (II/III layer and V layer), somatosensory cortex (II/III layer and V layer), *corpus striatum* (dorsal-lateral, dorsal-medial, and ventral), dentate gyrus, *Cornu Ammonis* region 3, entorhinal cortex, *substantia nigra pars compacta* and *reticulata*. Four images, at 20× for each area (one for each hemisphere at least in two different sections), were selected from the whole acquired sections (4 for each animal, two rostral sections for cortical and striatal areas, two caudal sections for nigral, hippocampal and entorhinal ones). The images were then deconvoluted and spatially calibrated using Fiji. For each image, a mean pixel signal intensity (in optical density; OD) inside the ROI was obtained and compared between the different areas. We analyzed at least 3–4 images per each area in each animal. We statistically tested whether subregional differences in α-synuclein intensity exist in the prefrontal cortex, somatosensory cortex, and *corpus striatum*. No subregional differences were detected; therefore, we decided to combine the data within each area.

#### 4.4.3. Immunofluorescence Procedure

Sections were permeabilized and blocked as described for immunoperoxidase histochemistry. They were next incubated for two nights in a mixture of primary antibodies—anti-α-synuclein S3062 and anti-α-tubulin T6074—with or without anti-synaptophysin 1. After rinsing with PBS, an incubation with the following secondary antibodies was performed for 75 min: donkey anti-rabbit Alexa Fluor^®^ 488 (Invitrogen, Waltham, MA, USA), donkey anti-mouse CF^®^ 568 (Biotium, San Francisco, CA, USA), both diluted 1:200 in BSA 0.1%. For the triple immunostaining, the slices were incubated with a mixture of donkey anti-rabbit Alexa Fluor^®^ 488 (1:200; Invitrogen), donkey anti-guinea pig CF^®^ 568 (1:200; Biotium) and biotinylated horse anti-mouse secondary antibody (Vector Laboratories) in BSA 0.1% for 75 min at room temperature, followed by an incubation with Alexa Fluor^®^ 647-conjugated streptavidin (1:200; Invitrogen) in PBS at room temperature for 2 h. Nuclei were stained using Hoechst 33342 (1:1000 in PBS; Sigma-Aldrich) for 15 min. Samples were mounted on coverslips using Mowiol^®^-DABCO (Sigma-Aldrich). Double immunostaining images (4 for each area) were acquired with a Nikon A1 laser scanning confocal microscope with a 40× magnification objective, while triple fluorescent labelling images were acquired by a Leica TCS SP8 scanning confocal laser at 40× magnification with an optical zoom 2. The specificity of primary antibodies was assessed by negative controls, e.g., omission of primary antiserum. In these cases, no specific staining was ever observed.

### 4.5. Proximity Ligation Assay (PLA)

The in situ PLA enables the detection of protein-protein interactions in intact tissues [72,73]. For the PLA procedure, we analyzed murine wild type and OlaHsd brain sections, and human paraffin embedded brain sections, containing *corpus striatum* using the Duolink assay kit (Sigma-Aldrich), according to the manufacturer’s instructions.

For the brightfield PLA procedure, after rehydration of the human brain slices, murine (WT and C57BL/6J OlaHsd) and human samples were then incubated with H_2_O_2_ for 20 min at room temperature and in 0.1% Triton X-100 in 1% BSA for 30 min at room temperature, sequentially followed by the mixture of: (i) the primary antibodies (for human sections: anti-α-synuclein antibody Syn211, S5566, anti-α-tubulin antibody, ab4074; anti-βIII-tubulin T8660; for murine sections: anti-α-synuclein S3062 and anti-α-tubulin T6074) incubated with 1% BSA for 1 h at 37 °C, then overnight at room temperature; (ii) the secondary antibodies donkey anti-mouse IgG conjugated with Duolink PLA MINUS oligonucleotides and anti-rabbit IgG secondary antibodies conjugated with Duolink PLA PLUS oligonucleotides diluted 1:5 in Duolink Antibody Diluent for 2 h at 37 °C; (iii) Duolink ligation solution (1:5) and ligase (1:40) for 1 h at 37 °C; (iv) Duolink amplification reagents (1:5) and polymerase (1:80) for 2 h at 37 °C; (v) Duolink detection solution (1:5) for 1 h at room temperature followed by the incubation of 3,3′ Diaminobenzidine as chromogen (DAB, Dako kit). The sections were then counterstained with hematoxylin and rapidly dehydrated before mounting with Eukitt^®^ (O. Kindler GmbH).

For fluorescent PLA experiments, vibratome brain sections from WT mice (*n* = 4), after incubation in 0.1% Triton X-100 in 1% BSA for 30 min at room temperature, were treated with the mixture of primary antibodies (anti-α-synuclein S3062, anti-α-tubulin T6074 and anti-synaptophysin) diluted in Duolink PLA diluent for 2 h at 37 °C, then overnight at room temperature. In order to verify PLA signal specificity, vibratome brain sections from WT mice were incubated with anti-βIII tubulin ab52901, anti-α-tubulin T6074 and anti-synaptophysin 1as positive control. All primary antibodies were incubated with 0.1% Triton X-100 and 1% BSA for 1 h at 37 °C, then overnight at room temperature. After washing, all samples were incubated with the mix of secondary antibodies conjugated with Duolink PLA oligonucleotides and ligase, as described for the brightfield PLA procedure. After that, sections were incubated with a solution of Duolink amplification reagent green (1:5), Duolink polymerase (1:80) and donkey anti-guinea pig CF^®^ 568 (1:200) at 37 °C for 2 h. Finally, Hoechst 33342 dye (1:5000) was used for nuclei counterstaining. The samples were mounted using Mowiol^®^-DABCO (Sigma). PLA labelled samples were examined both with a Nikon A1 laser scanning confocal microscope at 40× magnification (optical zoom 2) and with a Nikon microscope, equipped with CSI-W1 confocal scanner unit using a silicon-immersion 100× objective. Intensity profiles were obtained by the high-resolution images obtained with the spinning disk analyzed with the NIS elements AR software.

### 4.6. Colocalization Analysis

Confocal micrographs were analyzed with Fiji software. Identical parameters were used to acquire images for the same antigen, as previously described [74]. In brief, 4 nonoverlapping pictures were acquired in at least two different sections, so that double or triple immunolabelling and immunofluorescence PLA procedure were analyzed at least 4 fields per region in each animal. The degree of colocalization of different antigens was calculated by Manders’ coefficients, computed with the ImageJ JACoP plug-in [75]. For PLA-synaptophysin colocalization, a fixed ROI around each neuron was analyzed (at least 60 neurons for each mouse, *n* = 4 mice). In triple immunolabelling, the LASX software (Leica, Wetzlar, Germany) made it possible to obtain a colocalization mask for α-synuclein/α-tubulin signals. This mask was overlapped with the third signal, i.e., synaptophysin, and the degree of colocalization was calculated as the Manders’ coefficient. In this way, we evaluated the presence of α-synuclein/α-tubulin double signal in synaptic terminals identified by synaptophysin.

### 4.7. Electron Microscopy

To preserve the ultrastructure of the tissues, 3 C57BL/6J WT mice at P60 were perfused with 4 % paraformaldehyde and 0.2% glutaraldehyde in PB. Sections with prefrontal cortex, *corpus striatum* and *substantia nigra pars compacta* were selected for immunoperoxidase or immunogold pre-embedding immunohistochemistry reaction. For immunoenzymatic α-synuclein localization, sections were processed as described in the immunoperoxidase procedure paragraph, only avoiding Triton X-100 treatment (see [76] for details). For immunogold single and double labelling after aldehyde quenching with 0.2% NaBH_4_ in PBS for 30 min, sections were permeabilized with a mild treatment with ethanol (10%, 25%, 10% in PBS), rinsed with PBS and treated to block the unspecific interaction sites for 30 min with 1% BSA in PBS. Next, they were incubated overnight with one or the mixture of the primary antibodies anti-α-synuclein S3062 and anti-α-tubulin T6074 in 1% BSA at room temperature. This procedure was followed by rinsing with PBS and, after that, incubation with biotinylated goat anti-rabbit secondary antibody (Vector Laboratories; diluted 1:100 in 0.1% BSA in PBS) for 4 h. After washing, sections were treated overnight with streptavidin-gold 6 nm (Aurion; diluted 1:10 in 0.1% BSA in PBS). The next day, slices were washed three times with PBS and three times with PB 0.1 M and then transferred in new glass boxes and rinsed with double-distilled water. Immunogold reaction was intensified with silver enhancement, using R-GENT SE-EM kit (Aurion, Wageningen, The Netherlands) for 90 min. Following washes with double-distilled water and with PB, the sections were incubated with a goat anti-mouse secondary antibody conjugated with Ultra-Small gold particles (Aurion) diluted 1:30 in BSA 0.1%. Following rinsing in PB, the sections were post-fixed with 1% glutaraldehyde in PB for 1 h, then rinsed with PB and double-distilled water in new glass boxes. The silver enhancement process was then repeated, using R-GENT SE-EM kit (Aurion) for 90 min. In this way, double immunolabelling gold particles that stain α-synuclein would be intensified two times and have a larger size compared to gold particles staining α-tubulin. Finally, both sections, processed according to the immunoperoxidase and immunogold procedures, were osmicated, dehydrated and epoxy embedded as previously described [70]. After polymerization, small areas from prefrontal cortex, *corpus striatum* and *substantia nigra pars compacta* sections were cut with a razor blade and glued to blank resin blocks for sectioning with a Reichert-Jung ultramicrotome (Leica). Ultrathin sections (50–70 nm) collected on Cu/Rh grids were counterstained with lead citrate, or left unstained, and examined with a Zeiss LEO912AB electron microscope (Zeiss).

### 4.8. Statistical Analysis

Data are given as mean values ± standard error of the mean (SEM) and represent data from a minimum of three independent experiments. Mean values of each experiment were subjected to logarithmic transformation and analyzed with one-way Analysis of Variance (ANOVA), using the Bonferroni *post*-*hoc* test. Statistical analysis was run with the GraphPad Prism 9.0.0 software. Unless otherwise indicated, detailed statistics are given in the figure legends.

## Figures and Tables

**Table 1:** List of primary antibodies.

Primary Antibody	Epitope	Host Species	Application	Dilution	Source	Catalog Number
Actin	C-term fragment	Rabbit	WB	1:2000	Sigma- Aldrich St. Louis, MO, USA	A2066
α-synuclein	C-term human α-syn (aa 111–132)	Rabbit	WB	1:2000	Sigma- Aldrich St. Louis, MO, USA	S3062
			IF/EM	1:500		
			IHC	1:1500		
			PLA	1:100		
α-synuclein (clone Syn211)	C-term human α-syn (aa 121–125)	Mouse	IF	1:1500	Sigma- Aldrich St. Louis, MO, USA	S5566
			PLA	1:100		
α-tubulin (clone B-5-1-2)	C-term	Mouse	IF/EM	1:500	Sigma- Aldrich St. Louis, MO, USA	T6074
			PLA	1:50		
α-tubulin	N-term (aa 1–100)	Rabbit	PLA	1:50	Abcam, Cambridge, UK	ab4074
βIII-tubulin	C-term	Mouse	PLA	1:300	Sigma- Aldrich St. Louis, MO, USA	T8660
βIII-tubulin (clone EP1331Y)	C-term (within aa400)	Rabbit	PLA	1:250	Abcam, Cambridge, UK	ab52901
Synaptophysin1	Human synaptophysin1 (aa 301–313)	Guinea pig	IF	1:400	Synaptic Systems, Goettingen, Germany	101 004

## Data Availability

The datasets used and analyzed during the current study are available from the corresponding authors upon reasonable request.

## Supplementary Materials

Supplementary materials can be found at https://www.mdpi.com/article/10.3390/ijms22179153/s1.
