# Supplementary material for: The Association between α-Synuclein and α-Tubulin in Brain Synapses
Source: Int J Mol Sci. 2021 Aug 25;22(17):9153. doi: 10.3390/ijms22179153 (PMC8430732; doi:10.3390/ijms22179153)
Supplement: Supplementary file 1 [file ijms-22-09153-s001.zip › ijms-1296821-supplementary.pdf]

Supplementary Materials: Supplementary materials can be found at https://www.mdpi.com/article/10.3390/ijms22179153/s1.

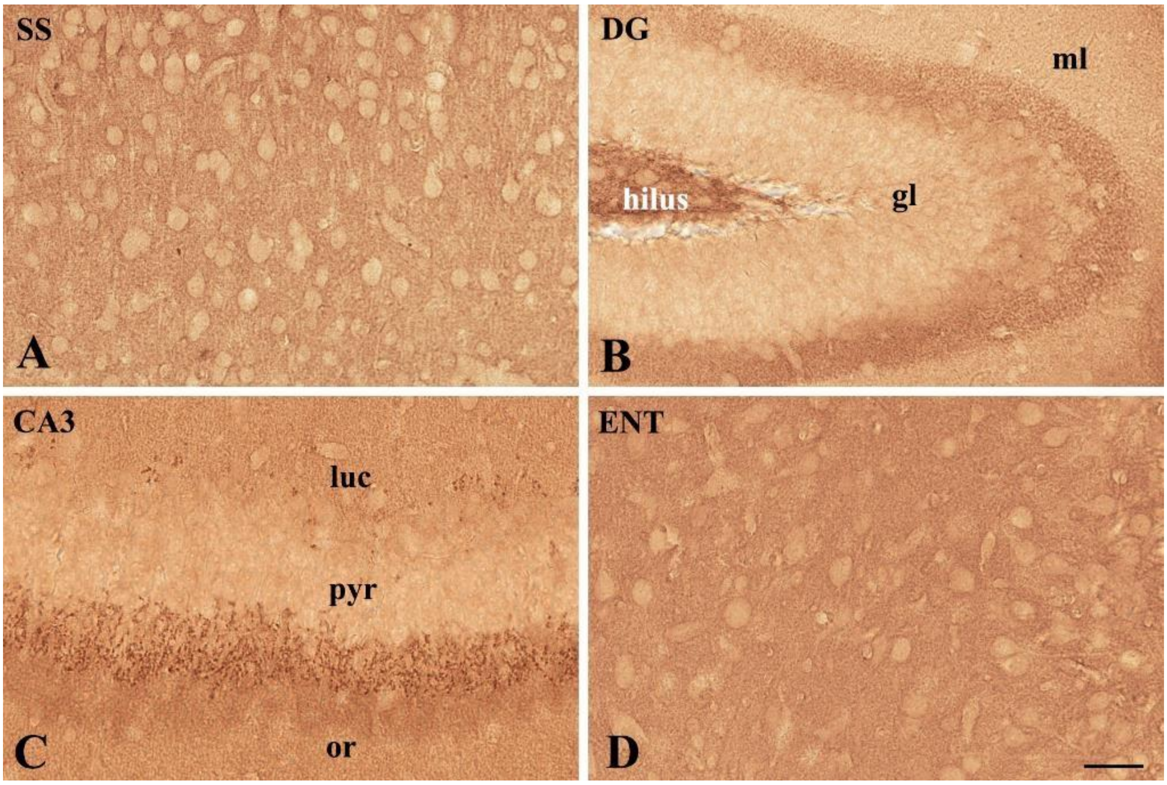

Figure S1. **α-Synuclein immunoperoxidase staining in coronal sections of WT mice brain.** Light microscope images of α-synuclein immunolabeling in somatosensory cortex (SS; **A**), dentate gyrus (DG; **B**), *Cornu Ammonis* region 3 (CA3; **C**) and entorhinal cortex (ENT; **D**). Several intense immunoreactive puncta were revealed both between granular (gl) and molecular (ml) layers of DG (B) and between pyramidal layer (pyr) and *stratum oriens* (or) of CA3 (**C**). luc: *stratum lucidum*. Scale bar: 40 μm.

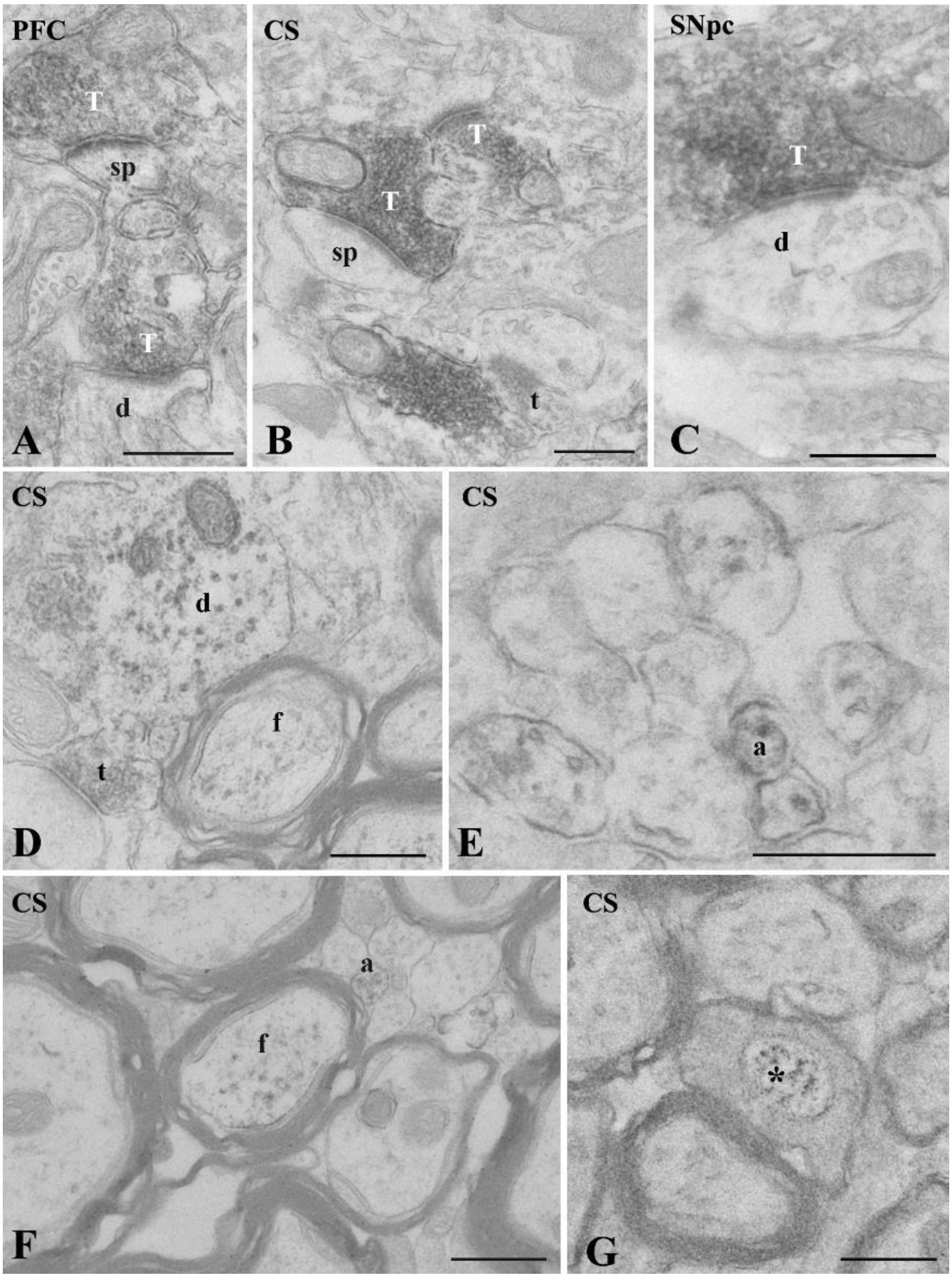

Figure S2. **Immunoperoxidase electron microscopy for α-synuclein in WT mice in prefrontal cortex (PFC; A),** ***corpus striatum*** **(CS; B, D, E, F, G) and** ***substantia nigra pars compacta*** **(SNpc; C).** Labelling consisted in the pre-embedding immunoperoxidase reaction product that resulted as a diffuse electron dense staining in proximity of the antigen, allowing the discrimination of the cellular compartments containing α-synuclein. In all the studied regions α-synuclein labelling was mainly at presynaptic level in association with synaptic vesicles (white T in **A-C**), but not all the synaptic boutons (t) appeared to be stained (**B, D**). α-Synuclein-positive synaptic terminals (white T) contacted mainly dendritic shafts (d) and dendritic spines (sp). In addition, the reaction product was present even in the dendritic (**D**) and in the axonal (**D-G**) compartments. In particular, myelinic fibers of large caliber (f in **D, F**), partially myelinated axons (asterisk in **G**) and amyelinic fibers of small caliber (a in **E, F**) displayed a faint immunoperoxidase staining. White T: α-synuclein-positive synaptic terminal; t: unlabeled synaptic terminal; sp: dendritic spine; d: dendrite; f: myelinic fiber; a: amyelinic small caliber axon; *=partially myelinated axon. Scale bars: 500 nm.

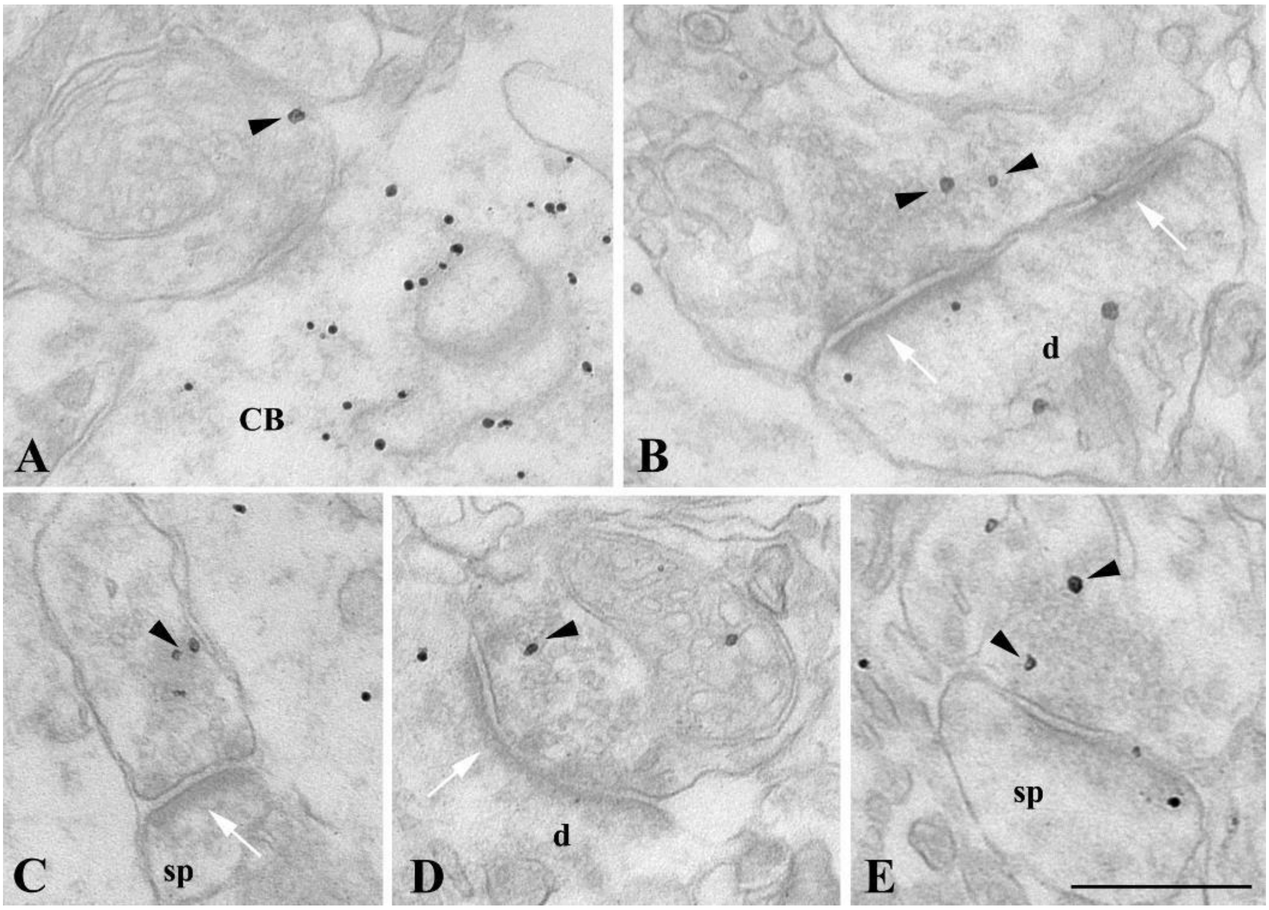

Figure S3. **Single α-tubulin pre-embedding silver-intensified immunogold labelling in WT murine *corpus striatum*.** A considerable amount of gold particles was observed in a neuronal cell body (CB in **A**), which was contacted by a small synaptic bouton showing one gold particle (arrowhead). In the striatal neuropil different vesicle-rich terminals were poorly immunolabelled (few gold particles, arrowheads) and made asymmetric (white arrows in **B, C, D**) or symmetric (**E**) synaptic contacts on distal dendritic processes (d in **B, D**) and dendritic spines (sp in **C, E**). Scale bar: 500 nm.

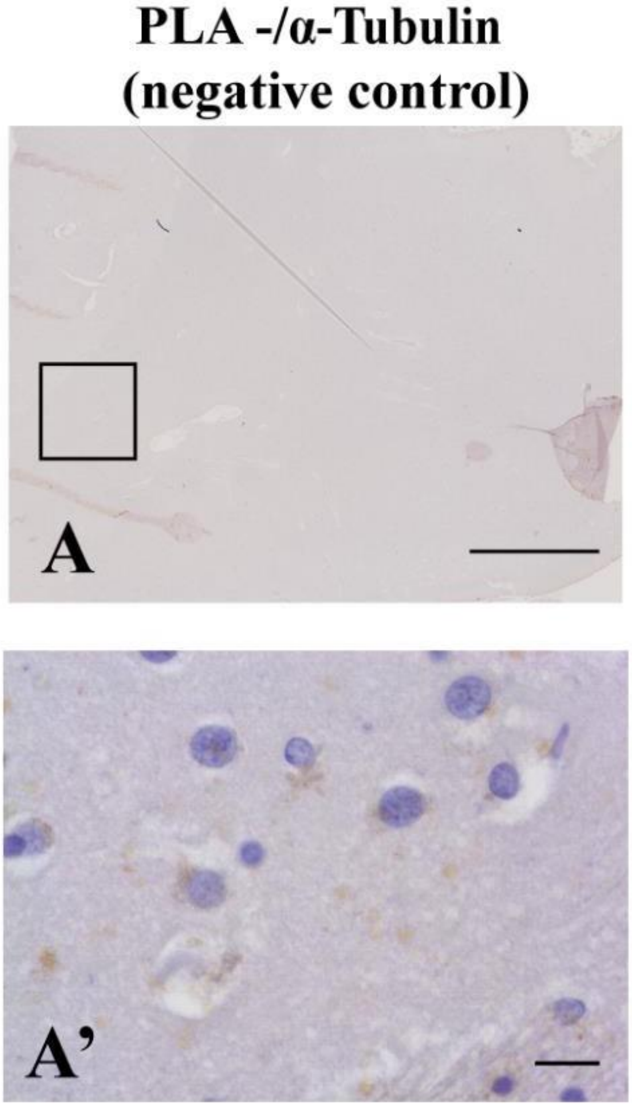

Figure S4. Negative control for brightfield PLA procedure in human *corpus striatum* and cerebral cortex. A: low magnification image negative control of PLA staining (brown), in which the primary antibody against α-Synuclein was omitted (-/α-Tubulin PLA negative control). **A′**: photomicrograph of the area selected with the black square in **A** at the level of the putamen. Nuclei are counterstained with hematoxylin (violet). Scale bars: 2.5 mm (**A**), 20 µm (**A′**).

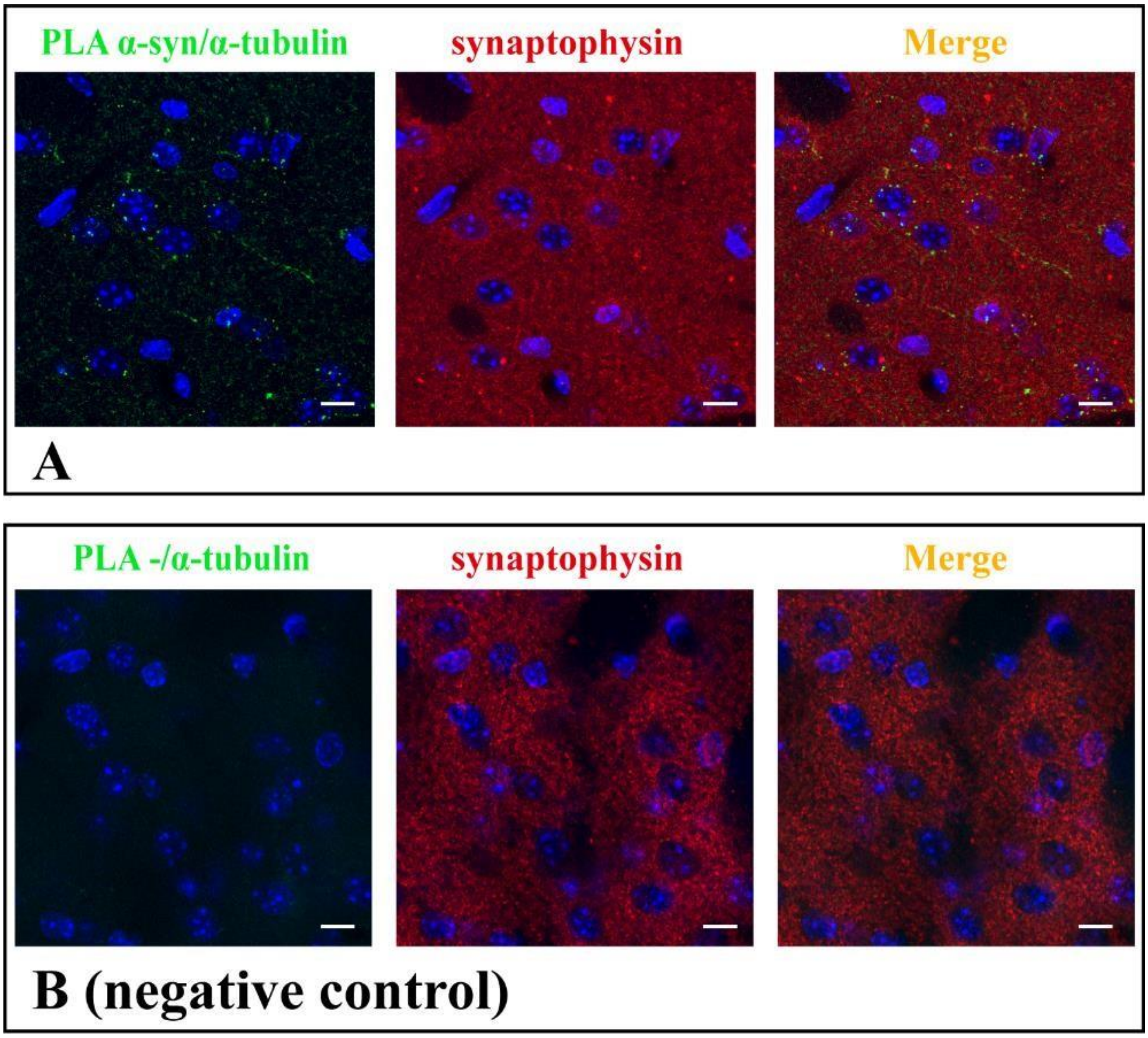

Figure S5. Confocal images of α-synuclein/α-tubulin PLA staining in mouse corpus striatum. A: from left to the right representative maximum projection of α-synuclein/α-tubulin PLA interaction (green), synaptophysin (red) and merge (all channels). **B:** from left to the right representative maximum projection of the negative control of the PLA staining in panel **A**, in which the primary antibody against α-synuclein was omitted. -/α-tubulin PLA (green), synaptophysin (red) and merge (all channels). Nuclei are counterstained with Hoechst (blue). Scale bars: 10 μm.

Supporting information

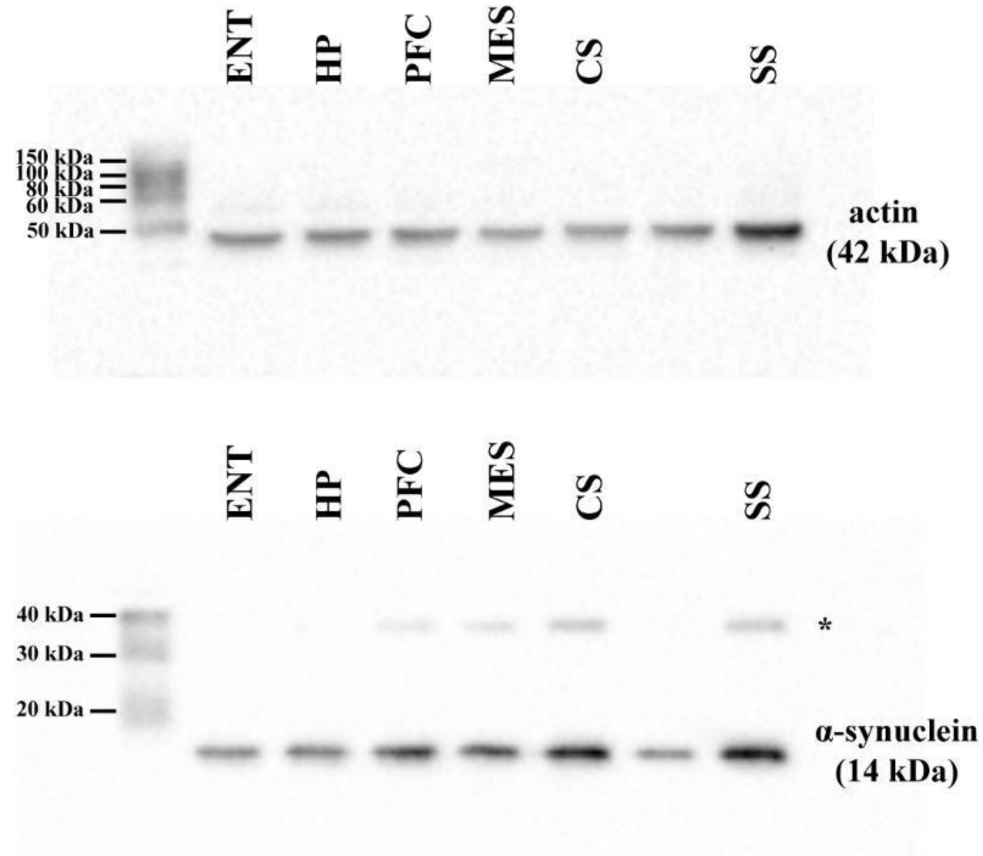

Original uncropped images of western blot analysis of α-synuclein levels in total lysates obtained from different regions of WT mouse brain. Uncropped images of western blot showing α-synuclein levels in total lysates of entorhinal cortex (ENT), *hippocampus* (HP), prefrontal cortex (PFC), ventral *mesencephalon* (MES), *corpus striatum* (CS), and somatosensorial cortex (SS). The whole membrane was cut between 40 kDa and 50 kDa and then separately treated only with anti-actin primary antibody (upper image) and only with anti-α-synuclein primary antibody (lower image). It is indicated with the asterisk (*) an α-synuclein aspecific band between 30 kDa and 40 kDa. Actin was used as loading reference.
